# Industrial Hygiene: Factory Nursing

**Published:** 1919-02-15

**Authors:** 


### THE LIFE OF A NURSE: ITS MORAL AND MATERIAL ADVANTAGES.

### II. Industrial Hygiene: Factory Nursing.

Factory nursing is a comparatively new branch of nursing and it offers many advantages and good prospects to women who are keenly interested in industrial conditions.

The factory nurse came into being as part of the Factory Welfare Department, which, since the outbreak of war, has come to be so universally recognised in any factory of note. Previous to the war, those factories recognising welfare departments—and they were few and far between—usually employed one officer only, and this lady—generally possessing either Red Cross or St. John's certificate—was considered capable to cope with any small illnesses or minor injuries likely to arise. Even now, in the smaller factories and in the less dangerous trades, the welfare officer can herself undertake the first-aid arrangements. But with the sudden and overwhelming advent of women as employees in munition and engineering firms, it was found absolutely necessary to employ a special nursing staff. Where deleterious substances have to be handled, such as the well-known "T. N. T.," a works doctor must also be in attendance, and the health staff sometimes assumes considerable dimensions. Since the passing of the Act of 1916 through the activities of the Home Office (Ministry of Munitions Welfare Dept.), when power was taken to require by order in any factory or group of factories that provision be made for various amenities such as protective clothing, drinking water, mess-rooms, first-aid, etc., etc., the trained nurse has been greatly in demand. So great has been the benefit of her ministrations on the general health of the workpeople that even when the war is over, she will almost certainly be installed as permanent officer in the majority of factories.

### Qualifications.

The conditions of training for nursing in factories are practically similar to those required for the other branches. A three years' course in a recognised hospital and the securing of her certificate of training is the *sine qua non* for a factory post, and if in addition the candidate has had a brief experience of health visiting, of hospital almoner work, she is practically certain to obtain a good post without difficulty.

The nurse has occasionally to undertake home-visiting of sick employees as part of her factory duties, therefore she should be instructed as to the routine of visits so that these may be undertaken with tact and sympathy. Should she be a qualified Sanitary Inspector her work will be of double value, and entitle her to act as adviser to the firm in all matters connected with the internal sanitary arrangements of the factory. Many trained nurses, if doubly qualified, *e.g.*, possessing either Health Visitors' or Sanitary Inspectors' certificate, take a post as Welfare Supervisor, where, in addition to the supervision of the nursing staff, they are entrusted with important administrative and advisory responsibilities.

A maternity training is of great value to a nurse dealing with large numbers of women. So many married women are now workers, that her guidance and supervision, especially when dealing with cases of pregnancy, are of great value, and in fact many firms now insist on the nurse appointed possessing her midwifery certificate. The nurse can play a large part in helping to maintain a high level of industrial efficiency by safeguarding the health of the future mothers and children and by carefully ensuring that any suggestion of fatigue or strain is at once alleviated.

To play an effective part in the management of the factory a nurse should, before taking up her duties, make herself acquainted with industrial conditions and the life inside the factory. Such a knowledge will help her to work smoothly with the various heads of departments, foremen, etc., and to avoid misunderstanding and bickering which are apt to arise when there is insufficient knowledge or co-operation on either side.

### Material and Moral Advantages.

Factory nursing is well paid, and what is also an undoubted attraction, there is no institution life. After her three years in hospital, it is often a great relief to a broad-minded woman to again be able to live at home or in private rooms. To always live by clockwork and routine and with a large number of people—although it has many compensations and advantages—is apt to become tiresome, and individuality and initiative are sometimes apt to suffer. The hours on duty, usually from 11 till 12 per day, are long, but compare favourably with present hospital conditions, and are in addition neither so tiring nor so exacting as hospital duty, and there is the additional compensation of free week-ends from Saturday noon until Monday morning. Night shifts have usually to be worked on alternate weeks or fortnights, but will probably be abolished after the war, when the Factory Acts relating to women's work are restored to their original condition.

Nurses seldom receive less than £100 per annum as a minimum, and very often lunch and tea are given gratis in the factory canteen, and uniform provided. Should the nurse be well qualified she will be able to command a much higher salary. A Welfare Supervisor practically never receives less than £150 and her salary may rise to £300 or even more.

Here is work of lasting and perennial value and interest for those who possess sound health, nerves, common sense, and a good digestion—all factors of success to any nurse—and we can imagine no more valuable work than ministering to the care of those who are building up our industries. After the war output will have materially to be increased if we are to hold our own as one of the premier nations of the world, and Labour will require to work under better health conditions than ever before and to receive her rightful share of the world's goods if she is to support Capital and to join hands with her in sympathy and understanding. It will be the part of the factory nurse to see that the material comfort of the worker lays the sure foundation to a more enlightened spiritual and mental development.

☛ The previous article appeared on February 8, p. 414.

